# Rediscovery of PHI-1/PPP1R14B: Emerging Roles of Cellular PP1 Signaling Mediated by the *PPP1R14B* Gene Product in Multiple Cancers and Beyond

**DOI:** 10.3390/biom15030344

**Published:** 2025-02-27

**Authors:** Masumi Eto

**Affiliations:** 1 Graduate School of Veterinary Science, Okayama University of Science, Imabari 794-8555, Ehime, Japan; m-eto@ous.ac.jp; 2 Faculty of Veterinary Medicine, Okayama University of Science, Imabari 794-8555, Ehime, Japan

**Keywords:** cancer diagnosis, prognostic assessment, protein phosphatase-1, cell signaling, proteostasis

## Abstract

PHI-1, encoded by *PPP1R14B*, regulates cellular protein phosphatase-1 (PP1) signaling and has emerged as both a biomarker and therapeutic target. Initially identified as a phospholipase-neighboring gene (PNG), PHI-1 is now known for its phosphorylation-dependent inhibition of PP1 holoenzymes, with bi-directional roles depending on its expression levels. Under physiological conditions, PHI-1 selectively regulates PP1 activity to maintain cellular homeostasis, whereas its pathological upregulation promotes oncogenic pathways, stabilizes tumor-promoting proteins, and modulates immune responses. This article explores PHI-1’s emerging role as a pan-cancer biomarker in parallel with emphasizing its physiological functions in signaling networks, smooth muscle contraction, cytoskeletal dynamics, and selective proteostasis. The mechanistic insights highlight PHI-1’s potential in precision oncology, offering opportunities for developing diagnostics and therapies that target its conditional functions.

## 1. Introducing PHI-1/PPP1R14B: Its Historical Journey into Cellular PP1 Regulation

### 1.1. Cellular Phosphatase Signaling in Physiology and Pathology

Reciprocal phosphorylation–dephosphorylation cycles determine cellular phosphorylation levels. Protein phosphatases play a vital role in controlling the response rate, intensity, duration, and sensitivity to physiological stimuli and pathological stressors. Protein phosphatase-1 (PP1) is one of the most abundant Ser/Thr phosphatase in eukaryotic cells and the amino acid sequence is highly conserved between yeasts and mammals [1,2,3,4]. In general, the catalytic subunits of PP1—PP1α, PP1δ(β), PP1γ1, and PP1γ2—possess a broad substrate specificity [1,2,3,4]. The interaction with PP1 regulatory subunits, involving over 100 gene products, confers specific cellular actions to the PP1 catalytic subunits [1,2,3,4]. In parallel, there are endogenous PP1 inhibitor proteins that mediate cellular stimuli into each PP1 holoenzyme, a complex of the PP1 catalytic and regulatory subunits [1,4,5]. To fully unravel signaling pathways governing cellular phosphorylation homeostasis, we must characterize cellular functions of each PP1 inhibitor protein in both preserving the physiological balance and contributing to pathological processes [6].

### 1.2. The PPP1R14 Family

**Figure 1:**
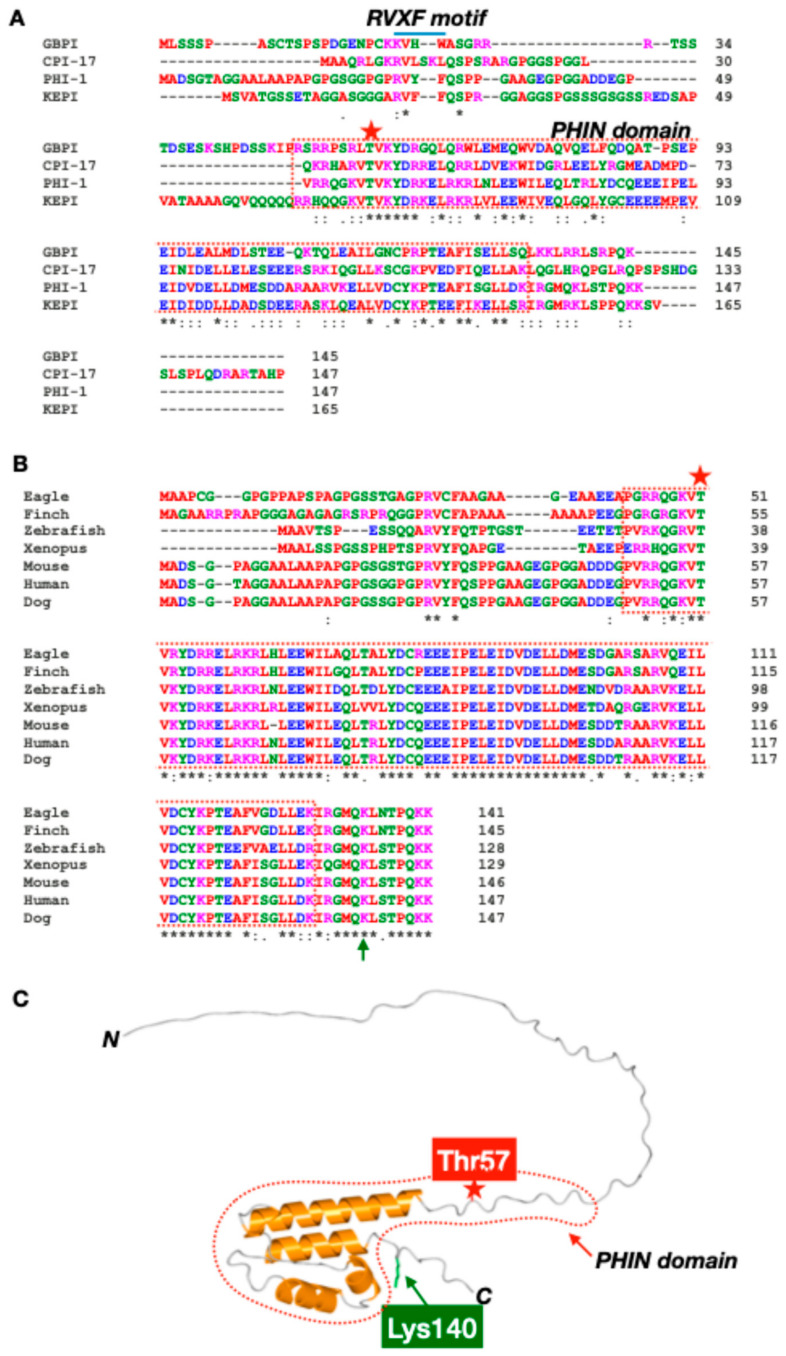
Structural insights into PHI-1. Sequence alignment of PPP1R14 family (**A**) and PHI-1 (**B**): FASTA data of PHI-1 proteins were obtained from NCBI Protein Database and subjected to analysis with Clustal Omega ver. 1.2.4 [18] for the sequence alignment. Color of each residue is based on their chemical properties: red (A, V, F, P, M, I, L, W), blue (D, E), green (S, T, Y, H, C, N, G, Q), and magenta (R, K). * indicates conserved residues. (**C**) Predicted 3D Architecture of human PHI-1: The 3D structure of unphosphorylated PHI-1 was predicted using AlphaFold 3.0 [19]. File conversion from CIF to PDB was conducted using PDBx/mmCIF software (https://mmcif.pdbj.org/converter/index.php?l=en, accessed on 9 January 2025). The 3D image was visualized and annotated using Waals 3.0 software (Altif Laboratories Inc., Tokyo, Japan). Red stars indicate Thr57, the phosphorylation site necessary for the PP1 inhibition, and green arrows indicate Lys140, the lactylation site regulating protein stability. Red dotted lines indicate the PHIN domain, conserved in the *PPP1R14* gene family.

The vertebrate genome encodes four members of the PPP1R14 gene family: CPI-17 (PPP1R14A), PHI-1 (PPP1R14B), KEPI (PPP1R14C), and GBPI (PPP1R14D) (Figure 1A). Each gene product shares the PHIN domain, —a structural element essential for phosphorylation-dependent PP1 holoenzyme inhibition—and plays distinct roles in cellular regulation [7,8,9,10,11]. In addition, except for CPI-17, a putative PP1 binding motif, RVXF, is found at the N-terminal domains of PHI-1, GBPI, and KEPI, although it remains unknown whether the site docks at PP1 catalytic subunits. Each member is suggested to recognize a specific subset of PP1 holoenzymes, probably due to direct interaction with regulatory subunits of PP1 holoenzymes [12].

### 1.3. Discovery and Characterization of PHI-1/PPP1R14B

PHI-1, encoded by *PPP1R14B* and renamed by the HUGO Gene Nomenclature Committee (HGNC:9057), is a selective endogenous inhibitor of PP1 holoenzymes, playing a multifaceted role in cellular regulation [8,11]. Initially identified as the phospholipase-C neighboring gene (PNG) during the mapping of the MEN1 locus on chromosome 11q13, PHI-1 was later redefined as a structural and functional regulator unrelated to MEN1-associated tumorigenesis [13,14,15]. Its ubiquitous expression and evolutionary conservation across species underscore its significance as a housekeeping gene involved in cellular homeostasis, although only limited numbers of PHI-1 orthologs have been detected in avian species [13,14,16].

### 1.4. Structure of PHI-1/PPP1R14B

Figure 1B highlights PHI-1’s evolutionary conservation, including its PHIN domain and the key phosphorylation site at Thr57. The phosphorylation of PHI-1 at Thr57, which corresponds to CPI-17’s Thr38, is a key event converting PHI-1 into a potent inhibitor of PP1 holoenzymes, with an IC50 of 2–30 nM [8]. AlphaFold predictions suggest an N-terminal intrinsically disordered domain preceding the PHIN domain (Figure 1, red dotted line) [11]. Based on the studies on CPI-17, the direct docking of the phosphorylated-PHIN domain at the active site of PP1 as a pseudo substrate is pivotal to PP1 regulation [11].

### 1.5. Structure–Function Relationship of PHI-1

Studies on zebrafish have provided evidence of the functional divergence within the *PPP1R14B* gene family. CPI-17 paralogs are primarily expressed in smooth muscle and neurons while PHI-1 paralogs are found in skeletal muscle and neurons [17]. Experiments with chimeric proteins have revealed that the PHIN domain determines inhibitory potency, reinforcing its critical role in functional specificity [17]. CPI-17 paralogs successfully rescue developmental phenotypes in zebrafish embryos, unlike PHI-1 paralogs, highlighting their selective roles in regulating PP1 pathways [17]. In addition to the PHIN domain, the N-terminal unstructured domain of CPI-17 is indispensable for regulating the myosin phosphatase in smooth muscle. The N-terminal region is unique among PHI-1, CPI-17, GBPI, and KEPI (Figure 1A), possibly contributing to their diverse roles in regulating a specific subset of cellular PP1 holoenzymes. Interestingly, the unstructured N-terminal domain of PHI-1 seems to be classified into three groups, birds, mammals, and anamniotes (fish and amphibians). PHI-1 may play distinct roles in PP1 holoenzyme regulation across vertebrates.

## 2. Deciphering PHI-1: Mechanisms Orchestrating Cellular Homeostasis in Normal States Encompassing Cytoskeletal Dynamics and Selective Proteostasis

**Figure 2:**
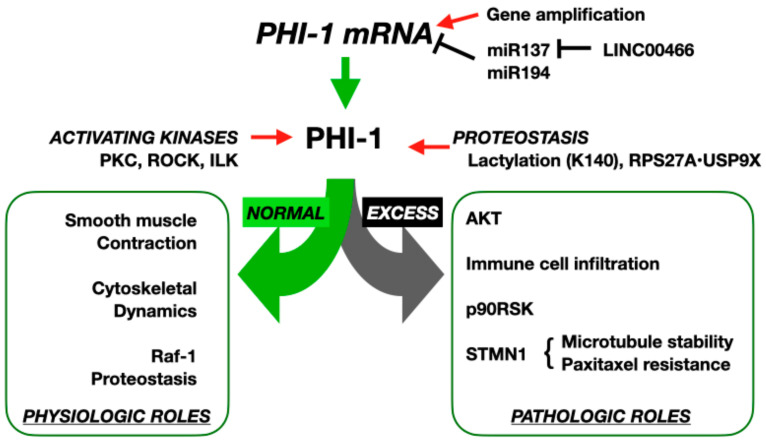
Physiological and pathological functions of PHI-1/PPP1R14B. At physiological expression levels, PHI-1 is activated by kinases such as PKC, ROCK, and ILK, leading to inhibition of PP1 holoenzymes that regulate smooth muscle contraction, cytoskeletal dynamics, and Raf-1 proteostasis. In pathological states, gene amplification or miR137 reduction by LIC00466 elevates PHI-1 mRNA; plus, Lys140 lactylation or interaction with RPS27A/USP9X increases PHI-1 stability. These post-transcriptional and post-translational mechanisms result in excessive PHI-1, driving AKT activation, immune cell infiltration, p90RSK activation, and STMN1-mediated microtubule stabilization, contributing to paclitaxel resistance in cancer cells.

Accumulating lines of evidence suggest that PHI-1 actions extend to key processes such as smooth muscle contraction [20,21,22,23], apoptosis [24,25], cell motility [26,27,28], metabolic regulation [29], and cytoskeletal dynamics [26] (Figure 2).

### 2.1. PHI-1’s Role in Regulating the Myosin Phosphatase and Smooth Muscle Contraction

PHI-1 was identified as a homolog of CPI-17, an endogenous regulator that selectively regulates the myosin phosphatase, a PP1 holoenzyme, and blood pressure [8,30,31]. The resemblance has driven extensive studies on PHI-1’s role in regulating smooth muscle contraction [20,21,22,23,32]. Recombinant PHI-1 thio-phosphorylated at Thr57 effectively inhibits endogenous myosin phosphatase activity, driving contraction in skinned smooth muscle fibers [20,22]. Among the kinases capable of phosphorylating Thr57, PKC, ROCK, and integrin-linked kinase (ILK) are prominent, with ILK demonstrating the highest specificity (Figure 2) [20,21,23]. In smooth muscle tissues such as the chicken gizzard and aorta, PHI-1 phosphorylation is induced by agonists like angiotensin II, thrombin, and a thromboxane analog, U-46619, in parallel with elevated myosin light chain phosphorylation [21,23]. This process is G-protein-mediated and inhibited by PKC or ROCK antagonists, highlighting the upstream regulation of PHI-1 by these kinases [21,22,23]. Upon stimulation, phosphorylated PHI-1 interacts with PP1, further supporting its role as a phosphorylation-dependent regulator of the myosin phosphatase (Figure 2) [22]. These findings demonstrate possible roles of PHI-1 as a phosphorylation-dependent regulator of the myosin phosphatase, linking its structural features and kinase-mediated activation to essential processes such as smooth muscle contraction and cytoskeletal regulation.

Although PHI-1 shares structural similarities with CPI-17, their functional profiles differ significantly. Unlike CPI-17, PHI-1 does not respond to phosphorylation by ZIPK/DAPK3, highlighting distinct activation pathways, although the sequence around the phosphorylation site is highly conserved (Figure 1A) [33]. Species-specific differences further illustrate the functional nuances of PHI-1. In chickens, where CPI-17 is absent, PHI-1 assumes a role of the myosin phosphatase inhibitor, partially compensating for the lack of CPI-17 [21,32]. PHI-1 is less effective in mediating the PKC-dependent Ca²⁺ sensitization of chicken aorta tissues, suggesting that functional limitations may occur due to a significantly slower rate of PHI-1 phosphorylation by PKC and with less potency compared to that of CPI-17 [32]. These differences clearly show unique roles of PHI-1 in PP1 regulatory pathways, reflecting its ability to adapt to diverse cellular and species-specific demands while complementing the functions of CPI-17.

### 2.2. PHI-1’s Tissue-Specific Expression and Subcellular Distribution

PHI-1 is ubiquitously expressed across tissues [8,13,34] whereas CPI-17 expression is mostly confined to smooth muscle tissues, particularly arteries [30]. The broader tissue distribution of PHI-1 suggests PHI-1’s involvement in additional physiological roles beyond smooth muscle regulation and pathological processes such as tumor progression [8,13]. The distinct roles of PHI-1 and CPI-17 are also supported by their cellular distributions, in which CPI-17 is found in the cytoplasm whereas PHI-1 is concentrated at juxtamembrane regions in intestinal smooth muscle cells [34]. The distinct phosphorylation pathways and broader distribution of PHI-1 point to its involvement in unique physiological processes, raising questions about its primary functions and evolutionary significance.

### 2.3. PHI-1’s Roles in Regulating Cell Migration and Cytoskeletal Reorganization

The ubiquitous expression pattern aligns with PHI-1’s classification as a housekeeping gene, essential for maintaining basal functions in normal cells, involving the regulation of cytoskeletal dynamics. Tountas et al. demonstrated that PHI-1 localizes to the trailing edge of migrating endothelial cells, in which PHI-1 is highly expressed [26]. The knockdown of PHI-1 impairs migration and enhances spreading by reducing retraction events, suggesting a key regulatory role in cytoskeletal dynamics and adhesion turnover. Because the retraction of the trailing edge of migrating cells is regulated by the myosin phosphatase [35], localized regulation by PHI-1 may drive normal cell migration (Figure 2). PHI-1’s coordination of multiple PP1 holoenzymes regulating cytoskeletal dynamics and adhesion turnover appears indispensable for efficient cell migration.

### 2.4. PHI-1’s Roles in Selective Proteostasis

Although knocking down PHI-1 in cancer cells reduced cell proliferation, HEK293 cells, whose PHI-1 expression levels are less than 50% of those in HeLa cells, exhibited the opposite response to siRNA-mediated knockdown [25]. PHI-1 knockdown led to a 7.2-fold increase in Raf-1 expression and a 21-fold increase in ERK1/2 phosphorylation under growth conditions, along with an elevated cell proliferation and a reduced apoptosis [25]. Treatment with tautomycin, a PP1 inhibitor, decreased Raf-1 levels, mimicking the effect of PHI-1. This indicates that PP1 activity modulates Raf-1 degradation [25]. The PP1–PHI-1 axis controls Raf-1 proteostasis as PHI-1-mediated PP1 inhibition affects Raf-1 protein turnover [25]. Interestingly, the regulation of proteostasis is specific to Raf-1, but not B-Raf or other kinases. The study highlighted PHI-1 as a selective regulator of Raf-1 proteostasis and downstream ERK1/2 signaling in non-cancer-derived cells characterized by a limited copy number (Figure 2) [25]. PHI-1 emerges as a multifaceted regulator of cellular PP1 pathways, orchestrating its roles in cytoskeletal dynamics, cell migration, and proteostasis to support normal cellular function and adapt to varying physiological and pathological conditions.

## 3. Rediscovering PHI-1/PPP1R14B: Bridging Molecular Mechanisms and Diagnostic/Prognostic Potential in Cancer

Following the exploration of PHI-1’s structural insights and its role in maintaining cellular homeostasis, we now shift focus to rediscovering PHI-1 in the context of cancer. Initially recognized for its regulatory function in normal cellular processes, accumulating lines of evidence have uncovered involvements of PHI-1 in multiple cancer types (Table 1). As discussed below, fluctuations in PHI-1 expression have been linked to advanced tumor stages, poor survival rates, and aggressive phenotypes. This section highlights how PHI-1 transitions from a precise regulator of cellular homeostasis to a potential oncogenic driver, setting the stage for understanding its complex role in cancer biology.

### 3.1. Mechanisms of Dysregulated PHI-1/PPP1R14B Expression in Cancer Cells

Most findings linking PHI-1 expression to cancer pathogenesis have emerged from bioinformatic analyses of large datasets, in parallel with genetic analysis techniques such as microarray assay (Table 1). As listed in Table 1, these analyses reveal consistent patterns of PHI-1 dysregulation across multiple cancer types, such as bladder urothelial carcinoma (BLCA), endometrial carcinoma (UCEC), prostate cancer (PRAD), glioblastoma multiforme (GBM), and triple-negative breast cancer (TNBC), indicating its broad relevance in cancer biology. PHI-1 dysregulation in cancer is driven by alterations at multiple levels—genetic, post-transcriptional, and post-translational—transforming it from a selective regulator to a broad-spectrum disruptor of PP1 signaling (Table 1).

#### 3.1.1. Gene Amplification and mRNA Elevation

PHI-1 gene amplifications, as reported by Deng et al. [36], are observed in cancers such as BLCA, PRAD, and liver hepatocellular carcinoma (LIHC), leading to elevated PHI-1 expression. This upregulation contributes to enhanced oncogenic signaling, promoting proliferation and tumor progression. The frequent amplification of the *PPP1R14B* gene is associated with poor survival outcomes, as observed in bladder, endometrial, and prostate cancers [36]. These amplifications also correlate with increased mRNA expression and adverse clinical outcomes, suggesting a dose-dependent oncogenic role that may amplify PP1 signaling disruptions, thereby promoting tumor progression. Using the databases of ICGC and GEO, Mosquera-Ogueira et al. mined a novel three-gene expression signature (with SCGB2A1, KLF4, and PHI-1) that predicts short survival in a small subset of chronic lymphocytic leukemia patients (Table 1) [37]. This signature, including PHI-1, operates independently of traditional prognostic markers, providing new insights into the molecular basis of aggressive disease [37]. Worley et al. also found the PHI-1 mRNA to be among the gene products that are elevated in endometriosis-associated ovarian clear cell carcinoma compared with benign endometriosis (Table 1) [38]. In prostate cancer, PHI-1 mRNA is among the top upregulated genes identified via the Oncomine analysis, though it was not detected in patient plasma samples, limiting its utility as a non-invasive biomarker [39].

#### 3.1.2. Post-Transcriptional Fluctuations

PHI-1 expression is further modulated post-transcriptionally. In glioblastoma (GBM) and lower-grade gliomas (LGG), Zhao et al. demonstrated that LINC00466 acts as a competitive endogenous RNA (ceRNA), suppressing miR-137 and thereby increasing PHI-1 expression, which is linked to temozolomide resistance [40]. In contrast, Huang et al. reported that miRNA-194 reduces PHI-1 expression, adding another layer of complexity. The presence of elevated miR-194 levels was identified as a prognostic biomarker for gastrointestinal cancers (gastric, colorectal, and liver cancers) through genome-wide association studies (Table 1) [41]. The upregulation of miR-194 in cancer cells significantly reduced the expression of the four target genes, including PHI-1, at both mRNA and protein levels, suggesting that miR194-induced PHI-1 downregulation occurs in proliferating gastrointestinal cancers [41]. This observation may relate to the discovery of PPP1R14B-AS, a lncRNA transcribed from the antisense strand of the PPP1R14B gene locus. This lncRNA enhances mitochondrial respiration in LIHC and LUAD [42], regulates breast cancer growth through ceRNA mechanisms [43], and contributes to a ferroptosis-related signature in uveal melanoma [44]. Notably, Yang et al. suggested that PPP1R14B-AS1 interferes with PHI-1 mRNA, emphasizing the need to differentiate their roles [42]. Although roles in PHI-1 signaling remains unclear, further investigations into PPP1R14B-AS1 may help clarify its distinct contribution compared to PHI-1 upregulation. This paradox in the findings of PHI-1 post-transcriptional regulation—where both its up- and downregulation contribute to cancer progression—suggests the presence of multiple PHI-1 targets within cancers and highlights the need for careful studies that consider the cancer type, genetic background, tumor microenvironment, and treatment conditions.

#### 3.1.3. Post-Translational Modifications (PTMs)

Lysine lactylation (Kla) has emerged as a key focus in cancer biology because elevated glycolysis due to the Warburg effect leads to increased lactate production, which can enhance lysine lactylation [45]. Proteomic analysis for cellular lactylation, lactose analysis, has revealed that PHI-1 is lactylated at Lys140 within the C-terminal loop region (Figure 1, green) [29]. The Kla modification is elevated in cervical cancer cells with poor prognosis compared to normal tissues (Table 1) [29]. The K140R mutant PHI-1, a Kla-null version, has shown a significantly reduced ability to promote cervical cancer cell proliferation, migration, and invasion compared to the wild-type protein [29]. This reduced activity is due to the decreased protein stability of the Kla-null mutant [29]. In addition to Kla modification, Liao et al. demonstrated that deubiquitination via USP9X stabilizes PHI-1, correlating with aggressive tumor behavior and chemoresistance [28]. Ribosomal protein S27a (RPS27A) recruits the deubiquitinase USP9X to stabilize PHI-1, preventing its proteasome-mediated degradation, leading to the post-translational upregulation of PHI-1 and paclitaxicel resistance [28]. These findings highlight that post-translational modifications, such as lysine lactylation and deubiquitination, are critical for PHI-1 stability and function, directly influencing cancer cell proliferation, metastasis, and drug resistance, making them potential targets for therapeutic intervention.

The dysregulation of PHI-1 expression—whether through gene amplification, post-transcriptional modulation, or post-translational modifications—consistently correlates with aggressive tumor phenotypes and poor clinical outcomes. Understanding the causes of PHI-1 up- or downregulation, its underlying mechanisms in tumor progression, and its impact on therapy resistance could uncover novel clinical benefits, including potential biomarkers for prognosis and new targets for cancer treatment. Further studies are needed to elucidate the comprehensive interplay between PHI-1 and other oncogenic pathways.

### 3.2. Pathophysiological Role of PHI-1/PPP1R14B in Cancer Cells

Interestingly, many of PHI-1’s molecular functions in normal cells—such as regulating cytoskeletal dynamics, protein stability, and cell proliferation—are hijacked in cancer to support tumor growth and metastasis.

#### 3.2.1. Proliferation and Survival

Miquel-Serra et al., in 2010, reported that the *PPP1R14B* transcript is downregulated in an established human melanoma cell line (MeWo) when a tumor suppressor, the V3 isoform of versican, is ectopically expressed (Table 1) [46]. The downregulation in PHI-1 mRNA reduces the cell proliferation rate and cell cycle progression, suggesting a role of PHI-1 in the phenotypic change induced by the V3 overexpression (Table 1) [46]. In cervical cancer, PHI-1 activates the Akt pathway to promote cell survival [24]. PHI-1 knockdown reduces proliferation and increases apoptosis in HeLa and HEC-1A cell lines, likely through a decrease in Akt phosphorylation levels, suggesting that PHI-1 upregulation potentially promotes proliferation and inhibits apoptosis by activating the Akt signaling pathway in cancer cells (Figure 2) [24]. In addition, in hepatocellular carcinoma (LIHC), PHI-1 stabilizes p90RSK, amplifying oncogenic signaling [47]. The knockdown of PHI-1 reduces proliferation and colony formation in hepatocellular carcinoma cell lines, supporting its role in promoting tumor growth. PHI-1 was co-precipitated with p90RSK, stabilizing its protein levels and enhancing phosphorylation at Ser363 and Ser380 [47]. The mutation of p90RSK phosphorylation sites (Ser363/380) blocks PHI1-mediated effects on cell viability and migration, supporting a model where the pathological expression of PHI-1 elevates p90RSK stability, amplifying the ERK signaling in cancer cells [47] (Figure 2). Thus, excess PHI-1 in cancer cells likely elicits aberrant Akt and ERK signaling through the pathological off-target inhibition of PP1 holoenzymes.

#### 3.2.2. Migration and Invasion

The in vivo assay using the shRNA KD mouse model revealed that PHI-1 enhances tumor growth, lung metastasis, and paclitaxel resistance [28]. PHI-1, stabilized through the RPS27A/USP9X axis, selectively forms a complex with PP1α and PP1γ1 isoforms and regulates stathmin 1 (STMN1), a microtubule-destabilizing protein in TNBC [28]. The PHI-1-mediated regulation of STMN1 decreases α-tubulin acetylation and microtubule stability, enhancing TNBC cell cycle progression and resistance to paclitaxel (Figure 2) [28]. By promoting microtubule depolymerization, STMN1 drives cytoskeletal remodeling essential for migration, invasion, and mitotic progression. Its overexpression is often associated with increased tumor aggressiveness and poor clinical outcomes in various cancers. Thus, the RPS27A/USP9X-PHI1-STMN1 axis is a critical driver of TNBC progression and paclitaxel resistance. Consistently, PHI-1 knockdown reduces cell migration and invasion in vitro, whereas its overexpression promotes these aggressive behaviors. In liver cancer models, PHI-1 depletion suppresses tumor growth and metastasis in xenograft assays [47]. Similarly, PHI-1 knockdown induces an elongated shape and impairs the migration of HeLa cells [26]. These findings suggest that PHI-1 regulates cancer cell migration and invasion, primarily through the RPS27A/USP9X-PHI-1-STMN1 axis, though additional pathways may be involved depending on the cancer type. A deeper characterization of PHI-1’s role in metastasis and therapy resistance is essential for developing targeted cancer therapies.

#### 3.2.3. Immune Modulation

PHI-1 is linked to immune cell infiltration patterns. In UCEC, He et al. [48] demonstrated that PHI-1 correlates with increased Th2 cell infiltration and reduced cytotoxic immune cell activity, suggesting an immunosuppressive tumor microenvironment. In kidney renal clear cell carcinoma (KIRC), PHI-1 expression is associated with CD8+ T cell infiltration and with the proliferation and migration of KIRC, implicating it in immune evasion strategies [27]. PHI-1 expression also positively correlates with the presence of myeloid-derived suppressor cells (MDSCs) in breast and liver cancers [36], suggesting that PHI-1 contributes to immune evasion mechanisms that may impact the efficacy of immunotherapies. A key unanswered question is how PHI-1 upregulation in cancer cells promotes immune evasion: by inducing immunosuppressive cytokines, altering metabolism, enhancing extracellular vesicle secretion, or modulating immune checkpoints. Further investigation is warranted to establish these pathways.

Despite its diverse functions, PHI-1 seems to consistently promote tumor aggressiveness. Its ability to regulate both intrinsic tumor behaviors and the tumor microenvironment positions it as a critical mediator in cancer pathophysiology. PHI-1’s dual roles—supportive in normal physiology but dysregulated in cancer—likely arise from multifaceted functions of cellular PP1 holoenzymes, illustrating how its regulatory mechanisms can be subverted, contributing to malignancy.

### 3.3. Clinical Relevance of PHI-1 in Cancer

PHI-1’s role as a cancer driver directly translates into its clinical implications, serving as both a biomarker for tumor aggressiveness and therapy resistance as well as a potential therapeutic target across multiple cancer types (Table 1).

#### 3.3.1. Diagnostic Potential

PHI-1 distinguishes malignant from normal tissues in cancers such as prostate adenocarcinoma (PRAD) and ovarian clear cell carcinoma (OCCC), enhancing diagnostic accuracy [38,39]. In a study, PHI-1 upregulation was further confirmed through mRNA and protein analyses, highlighting its potential in tissue-based diagnostics [38].

#### 3.3.2. Prognostic Significance

High PHI-1 expression correlates with poor survival in TNBC, UCEC, and glioblastoma (GBM), serving as a strong prognostic marker [28,40,48]. In UCEC, PHI-1 expression is significantly associated with an advanced tumor grade and stage, as well as with poor overall survival [48]. The same gene signature analysis in chronic lymphocytic leukemia (CLL) characterizes PHI-1’s prognostic significance independent of traditional markers [37]. To establish PHI-1 as a prognostic biomarker and a potential therapeutic target in tumor management, further investigation is needed to link its role in immune cell infiltration with tumor progression and patient survival.

#### 3.3.3. Predictive Biomarker

PHI-1 predicts therapy resistance in TNBC (paclitaxel resistance) and GBM (temozolomide resistance), offering a tool for personalized treatment strategies [29,40]. Functional assays have demonstrated that PHI-1 knockdown restores drug sensitivity in resistant cell lines, suggesting a potential target for overcoming chemoresistance [29,40]. Expanding PHI-1 as a pan-therapeutic marker requires further investigation to determine whether its upregulation contributes to chemoresistance in other tumor types, thereby potentially broadening its relevance in cancer therapy. Furthermore, although there are currently no reports linking PHI-1 mutations to pathological conditions, the accumulation of clinical data may reveal such associations in the future.

The evidence clearly establishes PHI-1 as a key driver of tumor progression, therapy resistance, and immune evasion, highlighting its clinical versatility as a diagnostic, prognostic, and predictive biomarker. PHI-1 has the potential to fill gaps in current cancer biomarker panels, particularly in predicting therapy responses where traditional markers fall short. As research advances, fully harnessing PHI-1’s clinical potential could open new avenues for cancer diagnostics and targeted therapies.

**Table 1:** List of tumors in which PHI-1 is up-/downregulated. Italics indicate tumors with downregulated PHI-1.

Tumor Name	Datasets	References
MeWo Melanoma (V3 Isoform of Versican)	OncoChip cDNA microarray (CNIO, Madrid, Spain)	[46]
Endometriosis-Associated Ovarian Clear Cell Carcinoma (OCCC)	Affymetrix Human Gene 1.1 ST Arrays via the GeneAtlas Fluidic Station	[38]
Chronic Lymphocytic Leukemia (CLL)	International Cancer Genome Consortium (ICGC, EGAD00010000875), GEO (GSE22762)	[37]
Prostate Cancer	Oncomine database	[39]
Bladder Urothelial Carcinoma (BLCA)	TIMER2.0	[36]
Breast Invasive Carcinoma (BRCA)	TIMER2.0	[36]
Cervical Squamous Cell Carcinoma and Endocervical Adenocarcinoma (CESC)	TIMER2.0, TCGA, GTEx, Human Protein Atlas (HPA), Kaplan–Meier Plotter	[24,36]
Cholangiocarcinoma (CHOL)	TIMER2.0	[36]
Colon Adenocarcinoma (COAD)	TIMER2.0, CPTAC	[36]
Diffuse Large B-cell Lymphoma (DLBCL)	GTEx	[36]
Esophageal Carcinoma (ESCA)	TIMER2.0	[36]
Glioblastoma Multiforme (GBM)	TIMER2.0, TCGA-GBM/LGG, GTEx database, qRT-PCR	[36,40]
Brain Lower-Grade Glioma (LGG)	GTEx, TCGA-GBM/LGG, qRT-PCR	[36,40]
Head and Neck Squamous Cell Carcinoma (HNSC)	TIMER2.0	[36]
Kidney Chromophobe (KICH)	TIMER2.0	[36]
Kidney Renal Clear Cell Carcinoma (KIRC)	TIMER2.0, TCGA, ICGC, GEO (GSE40435, GSE53757, GSM4630028), UALCAN, RT-qPCR, LinkedOmics, TISIDB, single-cell RNA-seq	[27,36]
Kidney Renal Papillary Cell Carcinoma (KIRP)	TIMER2.0	[36]
Liver Hepatocellular Carcinoma (LIHC)	TIMER2.0, TCGA-LIHC (Liver Hepatocellular Carcinoma), Human Protein Atlas, Label-Free Quantitative Proteomics, In Vitro and In Vivo Experimental Data	[36,47]
Lung Adenocarcinoma (LUAD)	TIMER2.0, CPTAC	[36]
Lung Squamous Cell Carcinoma (LUSC)	TIMER2.0	[36]
Ovarian Cancer (OV)	GTEx, CPTAC	[36]
Prostate Adenocarcinoma (PRAD)	TIMER2.0	[36]
Rectum Adenocarcinoma (READ)	TIMER2.0	[36]
Stomach Adenocarcinoma (STAD)	TIMER2.0	[36]
Thyroid Carcinoma (THCA)	TIMER2.0	[36]
Uterine Carcinosarcoma (UCS)	GTEx	[36]
Uterine Corpus Endometrial Carcinoma (UCEC)	TIMER2.0, CPTAC, TCGA, GEO (GSE17025), Human Protein Atlas (HPA), Clinical Samples, GTEx, Kaplan-Meier Plotter	[24,36,48]
*Gastrointestinal Cancer (GIC)*	TCGA, RNA-Seq data (GSE137070, GSE134308), Ago-HITS-CLIP-seq (GSE137071), CRISPR/Cas9 proliferation screening data (DepMap)	[41]
Triple-Negative Breast Cancer (TNBC)	FUSCC-TNBC cohort (Quantitative proteomics: n = 90 TNBC tissues, n = 72 adjacent normal tissues; RNA-seq: n = 360 TNBC tissues, n = 88 adjacent normal tissues), TCGA, METABRIC	[28]
Cervical Cancer	TCGA, CGCI, GSE44001, single-cell RNA-seq (GSE168652), proteomics (HeLa cells), TIMER2.0 (immune cell infiltration), lactylation-specific proteomics data	[29]

## 4. Decoding PHI-1/PPP1R14B Paradigm: A Balancing Act Between Cellular Homeostasis and Tumor Progression

As illustrated in Figure 2, PHI-1 acts as a dual-faceted regulator of PP1 signaling, with critical roles in both physiological and pathological states. Under physiological conditions, PHI-1 functions as a selective inhibitor for a subset of cellular PP1 holoenzymes, precisely modulating cytoskeletal dynamics, the proteostasis of key signaling molecules like Raf-1, and likely other PP1-driven functions. This fine-tuned regulatory capacity is vital for maintaining cellular homeostasis and supporting fundamental processes such as cell motility and proliferation.

The transition from physiological to pathological states, however, is characterized by PHI-1 upregulation that may lead to the non-specific inhibition of cellular PP1 holoenzymes and disrupt regulatory circuits in cellular phosphorylation signaling. This dysregulation triggers oncogenic signaling pathways, stabilizes tumor-promoting proteins, and contributes to immune evasion, collectively driving tumor progression in multiple cancer types. Furthermore, the discussion in this article has been based entirely on the assumption that PHI-1 regulates PP1 signaling. However, it remains possible that PHI-1 influences other systems beyond PP1, particularly under pathological conditions where its expression is upregulated. To fully understand the physiological and pathological roles of PHI-1, it is essential to identify its physiological and pathological targets.

The dual nature of PHI-1, with it functioning as both a precise regulator in healthy cells and a driver of oncogenesis under pathological conditions, positions it as a promising diagnostic marker and therapeutic target. While its upregulation is associated with tumor aggressiveness and poor prognosis, its controlled inhibition is essential for normal cellular functions. Future research should focus on unraveling mechanisms governing this transition and developing strategies to selectively modulate PHI-1 activity. By addressing these challenges, we must be able to deepen our understanding of cellular PP1 signaling and pave the way for precision medicine approaches targeting PHI-1 in cancer and beyond.

## Data Availability

No new data were created or analyzed in this study.

## Abbreviations

The following abbreviations are used in this manuscript:

PKC	Protein kinase C
ROCK	Rho-associated coiled-coil-containing protein kinase
ILK	Integrin-linked kinase
ZIPK/DAPK3	Zipper-interacting protein kinase/Death-associated protein kinase 3
USP9X	Ubiquitin specific peptidase 9 X-linked
AKT	AK strain transforming/protein kinase B
RSK	Ribosomal S6 kinase
